# Coronary microvascular dysfunction in post-PCI target vessels: a systematic review and meta-analysis of prevalence and associated outcomes

**DOI:** 10.3389/fcvm.2025.1620204

**Published:** 2025-08-04

**Authors:** Yunze Li, Jinlan Duan, Hongxu Liu, Sheng Lin, Hongkun Xu, Xiang Li

**Affiliations:** ^1^ Graduate School, Beijing University of Chinese Medicine, Beijing, China; ^2^ Beijing Hospital of Traditional Chinese Medicine, Capital Medical University, Beijing, China; ^3^ Aviation Health Medical Center, Air China Limited, Beijing, China; ^4^ Guang’anmen Hospital, China Academy of Chinese Medical Sciences, Beijing, China; ^5^ Department of Traditional Chinese Medicine, Lhasa People’s Hospital, Tibet Autonomous Region, China

**Keywords:** coronary microvascular dysfunction, percutaneous coronary intervention, prevalence, outcomes, meta-analysis

## Abstract

**Background:**

Coronary microvascular dysfunction (CMD) in post-percutaneous coronary intervention (PCI) target vessels is increasingly recognized as a critical determinant of adverse cardiovascular outcomes, yet its prevalence and prognostic implications remain poorly characterized. We conducted a systematic review and meta-analysis to determine the prevalence of CMD in post-PCI target vessels and its associated clinical outcomes.

**Methods:**

We conducted a systematic review and meta-analysis of observational studies using quantitative coronary physiological assessments to evaluate CMD in post-PCI target vessels. Databases (PubMed, Embase, Web of Science) were searched from inception to January 2025. The pooled Prevalence, multivariable-adjusted hazard ratio (HR) and 95% confidence interval (CI) for clinical outcomes were calculated using random-effects models.

**Results:**

A total of 21 observational studies involving 6,632 patients were included. The pooled prevalence of CMD in post-PCI target vessels was 41.66% (95% CI: 34.18%–49.34%). Subgroup analyses revealed numerical variations in CMD prevalence across assessment methods, sex, clinical diagnoses, and target vessels, though intergroup differences did not reach statistical significance (all *P* > 0.05). The pooled prevalence of CMD was numerically higher in females (46.22% vs. 36.73% in males), patients with acute coronary syndrome (42.37% vs. 36.04% in chronic coronary syndrome), and those assessed via non-wire-based methods (44.72% vs. 35.65% in wire-based methods). CMD prevalence was comparable across target vessels (left anterior descending artery: 37.34%, left circumflex artery: 38.50%, right coronary artery: 39.09%). Patients with post-PCI thrombolysis in myocardial infarction (TIMI) flow grade ≤2 exhibited higher CMD prevalence than those with TIMI flow grade 3, with a statistically significant difference (75.36% vs. 37.26%, *P* = 0.0012). CMD in post-PCI target vessels was independently associated with a 3.10-fold increased risk of major adverse cardiovascular events (95% CI: 2.06–4.67) and a 4.66-fold risk of cardiac death or heart failure readmission (95% CI: 3.13–6.93).

**Conclusion:**

CMD in post-PCI target vessels is prevalent (approximately 40%) and independently associated with a elevated risk of adverse cardiovascular outcomes. Standardized diagnostic criteria and targeted interventions are urgently needed to improve outcomes in this population.

**Systematic review registration:**

https://www.crd.york.ac.uk/PROSPERO/, PROSPERO CRD42025637496.

## 1 Introduction

Coronary microvascular dysfunction (CMD), defined as a clinical syndrome characterized by structural or functional abnormalities of the coronary microvasculature and microvascular obstruction leading to myocardial ischemia, has been increasingly recognized as a critical determinant of adverse cardiovascular outcomes, even following successful epicardial coronary revascularization (1–3). While percutaneous coronary intervention (PCI) effectively restores macrovascular blood flow in patients with coronary artery disease (CAD), emerging evidence suggests that persistent microvascular dysfunction in post-PCI target vessels may compromise myocardial perfusion, resulting in ischemia, cardiac dysfunction, and adverse ventricular remodeling (4, 5).

To date, the true prevalence of CMD in post-PCI target vessels and its long-term prognostic implications remain incompletely understood. Current investigations have reported that CMD in post-PCI target vessels is observed across nearly all diagnostic subtypes of CAD, including acute coronary syndrome (ACS) and chronic coronary syndrome (CCS), with substantial variability in prevalence ranging from 20% to 80% (6–10). This heterogeneity may arise from multiple factors. Some studies indicate that ACS patients exhibit a higher prevalence of post-PCI CMD compared to CCS patients, likely due to differences in target lesion composition and thrombotic burden (11, 12). Methodological disparities in assessments and study designs further contribute to this variability. Direct testing of coronary microvascular function is now considered necessary to confirm the diagnosis of CMD (13). Quantitative coronary physiological assessments have revealed that a substantial proportion of post-PCI CMD cases occur in vessels with angiographically normal flow, suggesting that studies relying on non-physiological assessments systematically underestimate the true prevalence of CMD in post-PCI target vessels (13–15). Conversely, some studies have overestimated CMD prevalence by failing to differentiate between pre-existing non-target vessel CMD and post-PCI target vessel CMD, thereby conflating distinct pathophysiological entities (16–18).

However, large-scale clinical investigations comprehensively covering CAD subtypes and utilizing quantitative coronary physiological assessments to evaluate post-PCI target vessel CMD are still lacking. To address this research gap, we conducted a systematic review and meta-analysis of observational studies employing quantitative coronary physiological assessments to evaluate CMD in post-PCI target vessels, aiming to determine the prevalence of CMD and its associated clinical outcomes. These findings provide novel insights into the true burden and clinical implications of CMD in post-PCI target vessels.

## 2 Methods

This systematic review was performed according to the Preferred Reporting Items for Systematic Review and Meta-Analysis (PRISMA) (19), and the protocol was registered at PROSPERO (No. CRD42025637496).

### 2.1 Search strategy

Studies that assessed CMD in post-PCI target vessels of patients after PCI were systematically searched in PubMed, Embase, and Web of Science from from inception through January 20, 2025. We used the following search terms: “percutaneous coronary intervention”, and “coronary microvascular dysfunction”. The detailed search strategy is provided in Supplementary Table S1.

### 2.2 Selection criteria

Inclusion criteria were as follows: (1) reported the prevalence of CMD based on quantitative coronary physiological assessments of post-PCI target vessels (15); (2) prespecified diagnostic criteria for CMD were clearly defined; (3) provided data exclusively comprising of patients undergoing PCI.

Exclusion criteria were as follows: (1) the study was a review, case report, conference abstract, protocol, or non-observational study; (2) duplicate publications; (3) data or full-text were not available; (4) non-English language publications; (5) the study used multiple measurements but did not report composite diagnostic outcomes.

It should be noted that, due to the lack of systematic pre-PCI CMD assessment in many studies, the included cases included both *de novo* post-PCI CMD and pre-existing CMD in target vessels.

### 2.3 Study selection and data extraction

Two investigators independently screened the titles and abstracts and removed irrelevant studies according to the inclusion and exclusion criteria. Two investigators extracted the following data: first author's name, study design, year of publication, geographic region, the characteristics of patients undergoing PCI, the method and cut-off value used to diagnose CMD, sample size, prevalence, multivariable-adjusted hazard ratio (HR) for clinical outcome, and relevant follow-up duration. The threshold of diagnostics tests used to define the presence of CMD were based on each individual study. Any inconsistencies were resolved by discussion with a third investigator.

### 2.4 Quality assessment

Two investigators independently assessed methodological quality using the “Joannagen Briggs Institute (JBI) Critical Appraisal Checklist for Studies Reporting Prevalence Data” (20). The checklist consists of nine questions (1): Was the sample frame appropriate to address the target population? (2) Were study participants recruited in an appropriate way? (3) Was the sample size adequate? (4) Were the study subjects and setting described in detail? (5) Was data analysis conducted with sufficient coverage of the identified sample? (6) Were valid methods used for the identification of the condition? (7) Was the condition measured in a standard, reliable way for all participants? (8) Was there appropriate statistical analysis? and (9) Was the response rate adequate, and if not, was the low response rate managed appropriately? Each item has four options: yes, no, unclear, or not applicable. An overall score was used to reflect the number of questions with an option of “yes”.

### 2.5 Statistical analysis

To mitigate the risk of duplicate patient inclusion across overlapping studies, we systematically retained the study with the largest sample size for each meta-analysis when multiple publications potentially shared identical or overlapping populations (21). In cases of equivalent sample sizes, studies using index of microcirculatory resistance (IMR) or coronary flow reserve (CFR) were prioritized. To stabilize variance in proportion data, prevalence estimates were transformed using the Freeman-Tukey double arcsine method prior to pooling. Between-study heterogeneity was quantified via *τ*^2^ for variance estimation and *I*^2^ statistic for inconsistency assessment, with *I*^2^ > 50% indicating substantial heterogeneity. A random-effects model was applied to account for anticipated variability across studies, given the expected clinical and methodological diversity among included studies. The multivariable-adjusted hazard ratio (HR) and 95% confidence interval (CI) were used to pool clinical outcomes. Subgroup analyses were conducted according to the following: (1) assessment methods: non-wire-based and wire-based; (2) sex: male and female; (3) diagnosis: ACS and CCS; (4) target vessels: left anterior descending artery (LAD), left circumflex artery (LCX), and right coronary artery (RCA); (5) post-PCI thrombolysis in myocardial infarction (TIMI) flow grade: TIMI flow grade 3 and TIMI flow grade ≤2. Meta-analyses were pooled using the R Software (version 4.4.2 R Project for Statistical Computing, Vienna, Austria) and the “meta” R package (22). *P* value <0.05 was considered statistically significant.

## 3 Results

### 3.1 Literature search

**Figure 1:**
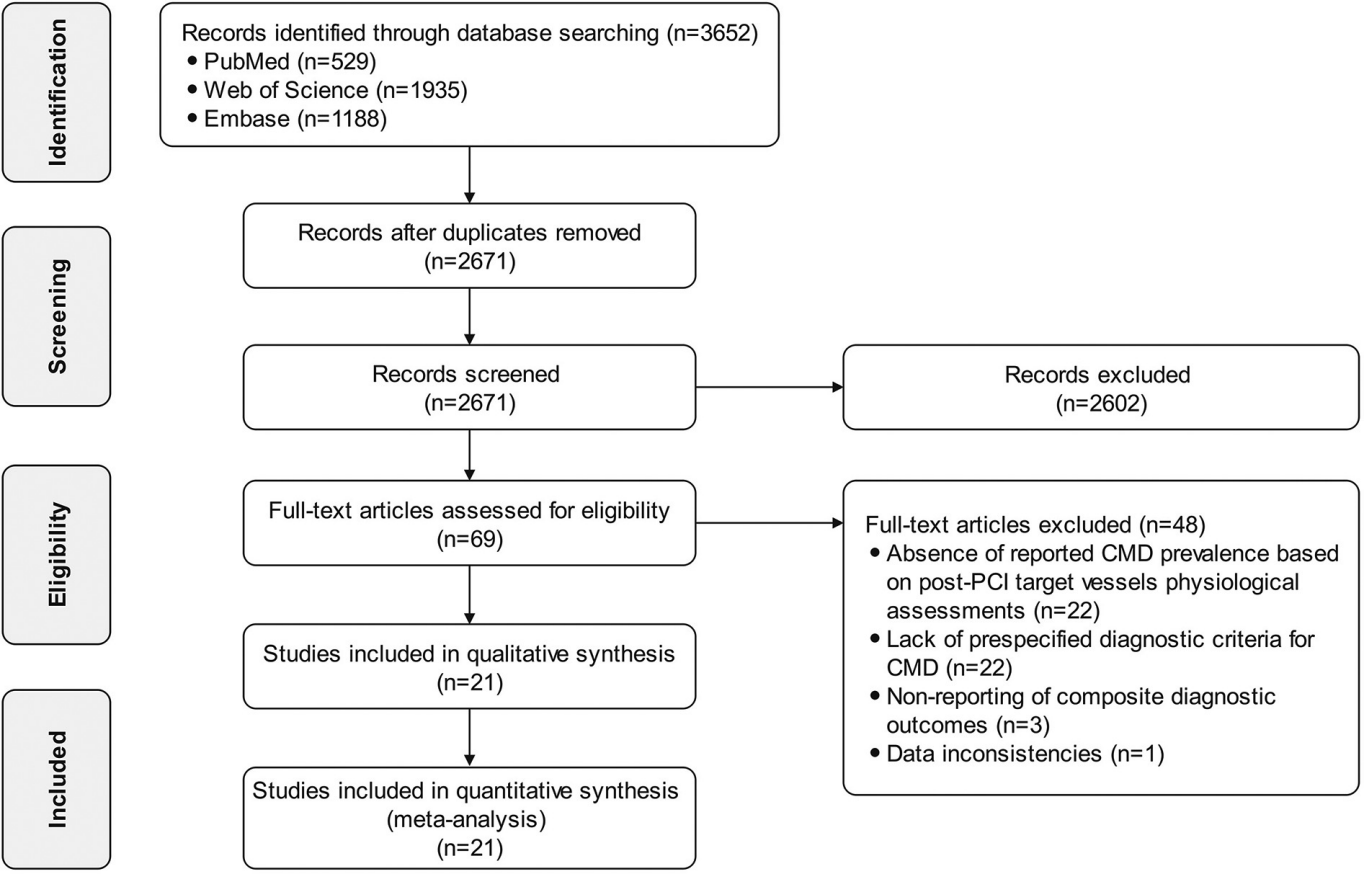
The study selection flowchart.

A total of 3,652 records were identified through systematic database searches, comprising 529 from PubMed, 1,935 from Web of Science, and 1,188 from Embase. After removing 981 duplicates, 2,671 records proceeded to title/abstract screening. Of these, 2,602 records were excluded due to irrelevance to CMD assessment in post-PCI target vessels. Subsequently, 69 full-text articles were assessed for eligibility. Ultimately, 21 studies (4, 5, 9, 10, 23–39) met all inclusion criteria and were incorporated into both qualitative synthesis and quantitative meta-analysis. The study selection process is detailed in Figure 1.

### 3.2 Characteristics of the included studies

The 21 included studies were conducted across diverse geographic regions, including France (34), China (5, 9, 23, 26–29, 33, 35), Lithuania (4, 36–38), Australia (10, 25), Belgium (10, 25), Japan (10, 25), the United States (10, 25), the United Kingdom (31), Germany (32), Serbia (39), and Korea (24, 30). These studies comprehensively covered diagnostic subtypes of CAD. All studies utilized coronary physiological assessments to diagnose CMD in post-PCI target vessels, employing either wire-based or non-wire-based methods. 9 studies (4, 29, 32, 33, 35–39) were prospective, and 12 were retrospective (5, 9, 10, 23–28, 30, 31, 34). 7 studies were multicenter investigations (5, 10, 24–27, 35). 14 studies (4, 5, 10, 23–27, 29, 31, 34, 36–38) reported post-PCI follow-up, with durations ranging from 30 days to 4.2 years (median). Notably, 4 single-center studies from Lithuania (4, 36–38) and 2 multicenter studies from Australia, Belgium, Japan, and the United States (10, 25) utilized overlapping populations. The characteristics of the included studies are presented in Table 1.

**Table 1:** Characteristics of the included studies.

Author	Region	Study design	Diagnosis	Total	CMD, n (%)	Criteria	Method	Follow-up
Caullery et al. (34)	France	retrospective	STEMI	178	72 (40%)	AngioIMR > 40	measured with angiography after nitroglycerin administration	2.9 years (median)
Li et al. (28)	China	retrospective	STEMI	418	157 (38%)	AccuIMR > 40	measured with angiography	NR
Aldujeli et al. (36)	Lithuania	prospective	STEMI	210	57 (27%)	IMR ≥ 25 or CFR < 2 with FFR ≥ 0.8	measured invasively with pressure sensor/thermistor-tipped guidewire after nitroglycerin and adenosine administration	1 year
Chen et al. (27)	China	retrospective, multicenter	NSTEMI	2212	513 (23%)	Angio-IMR ≥ 23	measured with angiography	2 years
Tian et al. (29)	China	prospective	STEMI	134	78 (58%)	AMR ≥ 2.5 mmHg·s/cm	measured with angiography	30 days
Zhang et al. (35)	China	prospective, multicenter	STEMI	1258	620 (49%)	AMR > 250 mmHg·s/m	measured with angiography	NR
Cui et al. (9)	China	retrospective	AMI	53	42 (79%)	MFR < 2 with residual stenosis < 50%	measured with SPECT after adenosine administration	NR
Tsai et al. (37)	Lithuania	prospective	STEMI	210	56 (27%)	MRR < 3	derived from CFR and FFR, measured invasively with pressure sensor/thermistor-tipped guidewire after adenosine administration	1 year
Aldujeli et al. (4)	Lithuania	prospective	STEMI	210	57 (27%)	IMR ≥ 25 or CFR < 2 with FFR > 0.8	measured invasively with pressure sensor/thermistor-tipped guidewire after nitroglycerin and adenosine administration	1 year
Wang et al. (23)	China	retrospective	STEMI	506	215 (42%)	AMR ≥ 250 mmHg·s/m	measured with angiography	1 year
Aldujeli et al. (38)	Lithuania	prospective	STEMI	210	57 (27%)	IMR ≥ 25 or CFR < 2	measured invasively with pressure sensor/thermistor-tipped guidewire after nitroglycerin and adenosine administration	1 year
Nishi et al. (25)	Australia, Belgium, Japan, USA	retrospective, multicenter	CCS	505	137 (27%)	IMR ≥ 25 with FFR > 0.8	measured invasively with pressure sensor/thermistor-tipped guidewire after nitroglycerin and adenosine administration	4 years (median)
Tang et al. (26)	China	retrospective, multicenter	STEMI	186	88 (47%)	IMRangio > 40	measured with angiography	2 years
Kotronias et al. (31)	UK	retrospective	STEMI	262	126 (48%)	NH IMRangio > 43	measured with angiography	4.2 years (median)
Kang et al. (24)	Korea	retrospective, multicenter	AMI	116	26 (22%)	IMR > 40	measured invasively with pressure sensor/thermistor-tipped guidewire after nitroglycerin and adenosine administration	40 months (median)
Dai et al. (5)	China	retrospective, multicenter	CCS	138	45 (33%)	Angio-IMR ≥ 25.1	measured with angiography	28 months (median)
Yang et al. (30)	Korea	retrospective	UA, CCS	39	9 (23%)	IMR ≥ 25	measured invasively with pressure sensor/thermistor-tipped guidewire after nitroglycerin and adenosine administration	NR
Nishi et al. (10)	Australia, Belgium, Japan, USA	retrospective, multicenter	CCS	572	148 (26%)	IMR ≥ 25	measured invasively with pressure sensor/thermistor-tipped guidewire after nitroglycerin and adenosine administration	4 years (median)
Trifunovic et al. (39)	Serbia	prospective	STEMI	96	39 (41%)	CFR < 2	measured with TDE after adenosine administration	NR
Werner et al. (32)	Germany	prospective	CCS	42	23 (55%)	CFVR < 2	measured invasively with Doppler guidewire after adenosine administration	NR
Qian et al. (33)	China	prospective	AMI, UA, CCS	212	137 (65%)	CFVR < 3	measured invasively with Doppler guidewire after nitroglycerin and adenosine administration	NR

AMI, acute myocardial infarction; AMR, angiography-derived microcirculatory resistance; CAD, coronary artery disease; CCS, chronic coronary syndrome; CMD, coronary microvascular dysfunction; CFR, coronary flow reserve; CFVR, coronary flow velocity reserve; FFR, fractional flow reserve; IMR, index of microcirculatory resistance; MFR, myocardial flow reserve; MRR, microvascular resistance reserve; NH, non-hyperemic; NSTEMI, non-ST-elevation myocardial infarction; NR, not reported; SPECT, single photon emission computed tomography; STEMI, ST-elevation myocardial infarction; TDE, transthoracic Doppler echocardiography; UA, unstable angina.

### 3.3 Methodological quality of the included studies

19 studies (4, 5, 9, 10, 23–29, 31, 33–39) with scores above 7 points indicated that most studies were of a high quality (21). 2 studies (30, 32) scored 6 points. 9 studies (4, 5, 10, 24, 25, 34, 36–38) did not report the number of patients lost to follow-up, and 7 studies (9, 28, 30, 32, 33, 35, 39) were not applicable (absence of follow-up). 4 studies (9, 30, 32, 39) did not have sufficient sample size. 7 studies (24, 30, 32, 33, 35, 36, 38) did not report the demographic and clinical characteristics of CMD patients in detail. The detailed assessment results are shown in Supplementary Table S2.

### 3.4 Meta-analysis

#### 3.4.1 Prevalence of CMD in post-PCI target vessels

To mitigate duplication bias, we included only studies with the largest sample sizes in the meta-analysis. Specifically, among 4 single-center (4, 36–38) studies and 2 multicenter studies (10, 25) that utilized overlapping populations, we prioritized those with the highest enrollment.

**Figure 2:**
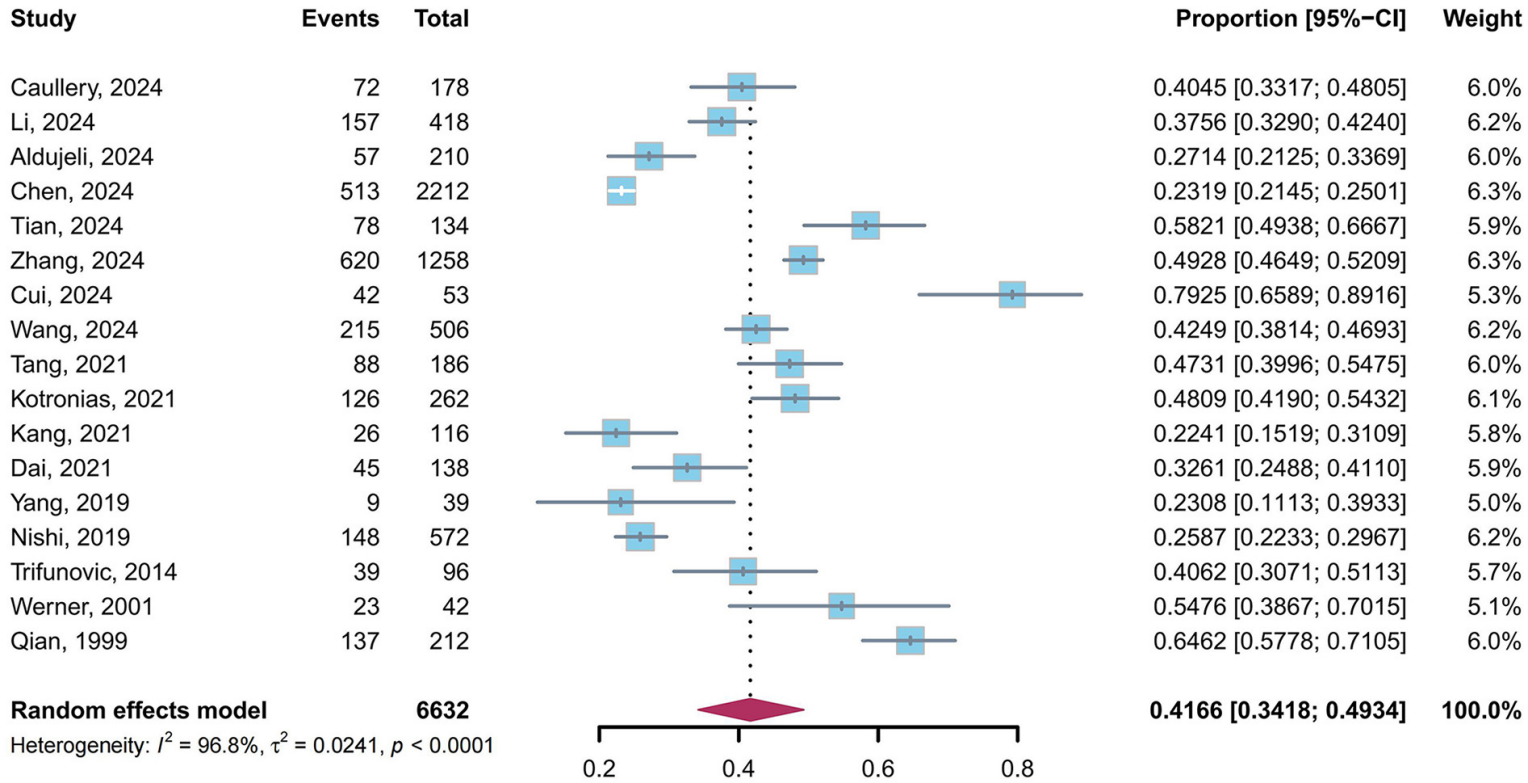
Prevalence of CMD in post-PCI target vessels.

We performed a pooled analysis of 17 studies (5, 9, 10, 23, 24, 26–36, 39) comprising 6,632 patients. The results of the random-effects model (*I*^2^ = 96.8%) showed that 41.66% (95% CI: 34.18%–49.34%) of post-PCI patients exhibited CMD in target vessels (Figure 2).

#### 3.4.2 Sensitivity analysis and publication bias

**Figure 3:**
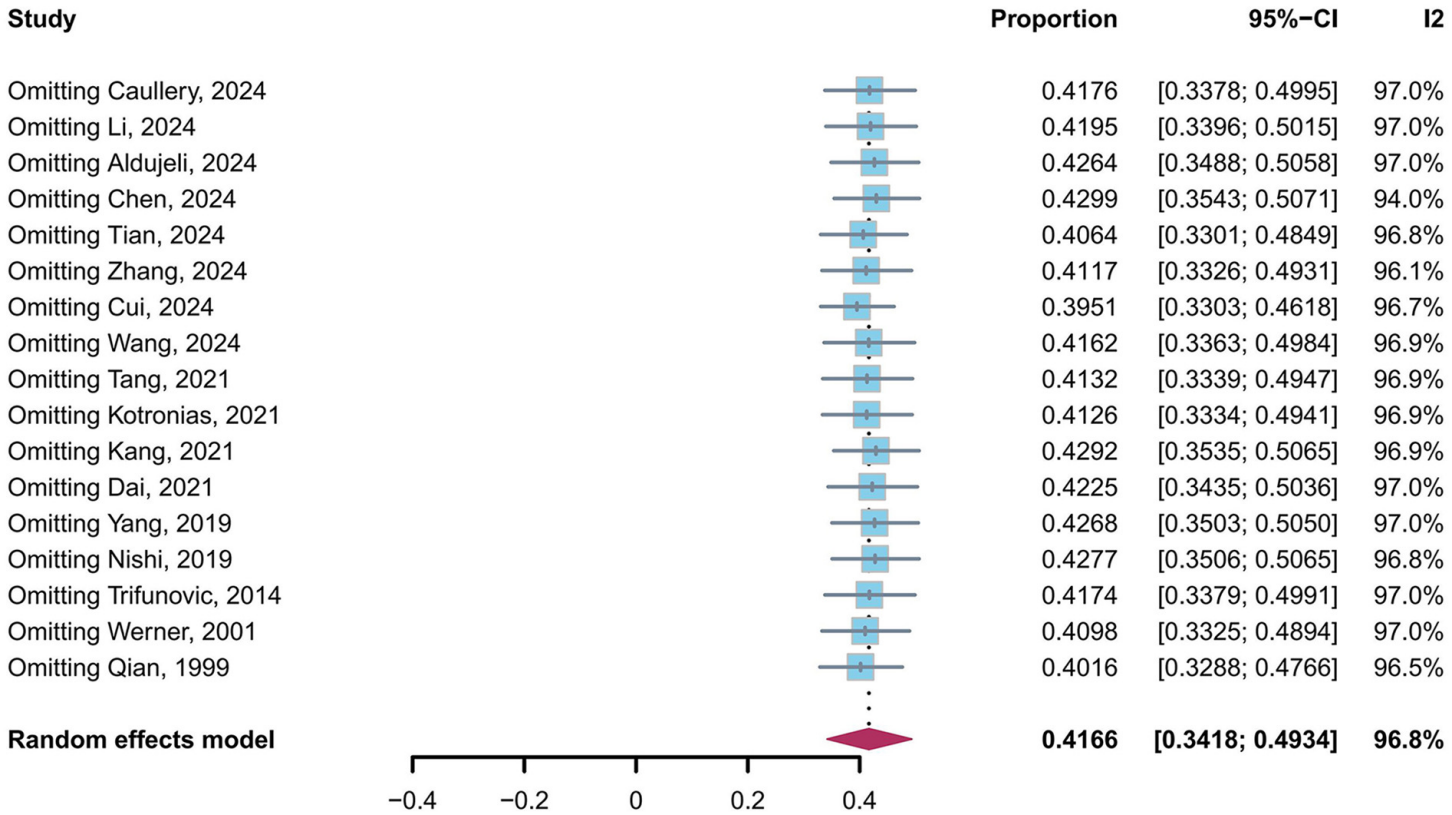
Sensitivity analysis of CMD in post-PCI target vessels prevalence.

A leave-one-out sensitivity analysis confirmed the robustness of the pooled prevalence estimate (Figure 3). The prevalence of CMD in post-PCI target vessels remained stable, ranging from 39.51% (95% CI: 33.03%–46.18%) to 42.99% (95% CI: 35.43%–50.71%), with no single study altering the estimate beyond this range. Despite substantial heterogeneity (*I*^2^ = 96.8%, *τ*^2^ = 0.0241, *P* < 0.0001), the consistency across sensitivity analyses supports that approximately 40% of post-PCI patients exhibit CMD in target vessels. Egger's tests were performed for the prevalence of CMD in post-PCI target vessels, and the results revealed that no publication bias was observed (*t* = 1.76, *p* = 0.10).

#### 3.4.3 Subgroup analysis

**Figure 4:**
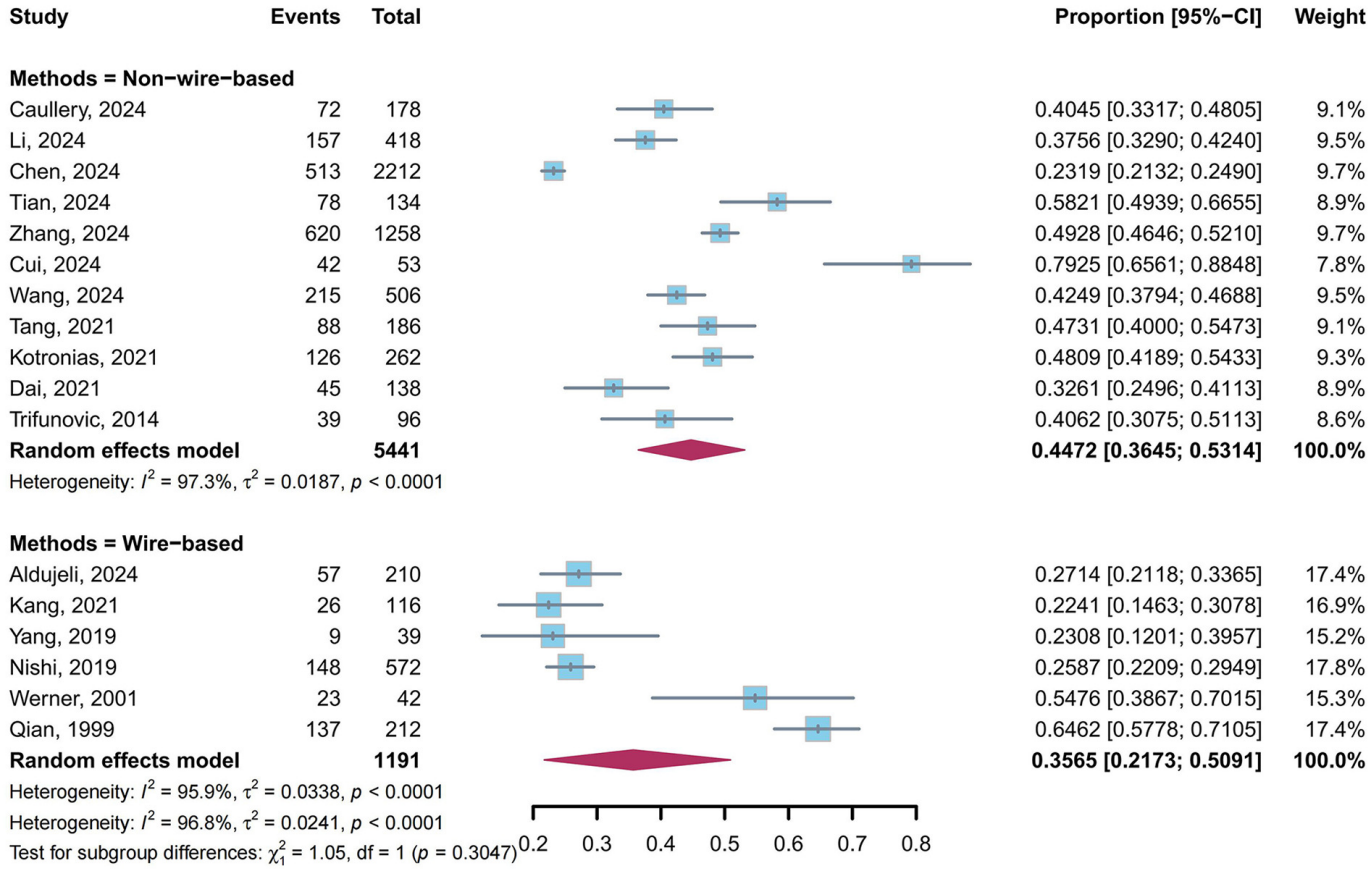
Prevalence of CMD in post-PCI target vessels: non-wire-based vs. wire-based subgroups.

A total of 11 studies (5, 9, 23, 26–29, 31, 34, 35, 39) utilized non-wire-based methods, and 6 studies (10, 24, 30, 32, 33, 36) employed wire-based methods to evaluate the prevalence of CMD in post-PCI target vessels. Our meta-analysis showed that the pooled prevalence of CMD was 44.72% (95% CI: 36.45%–53.14%, *I*^2^ = 97.3%) in non-wire-based methods and 35.65% (95% CI: 21.73%–50.91%, *I*^2^ = 95.9%) in wire-based methods, with no statistically significant difference between subgroups (*P* = 0.30) (Figure 4).

**Figure 5:**
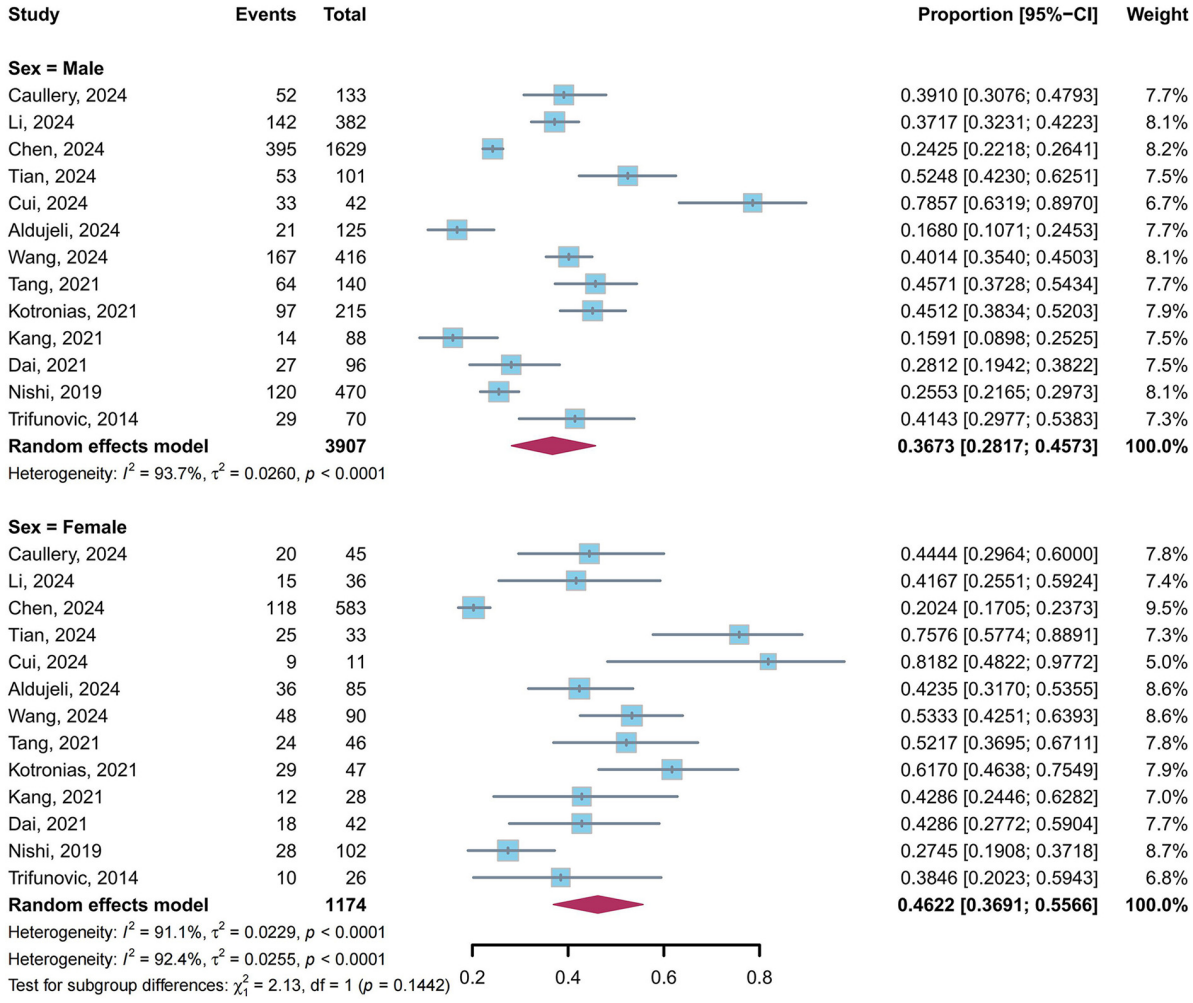
Prevalence of CMD in post-PCI target vessels: male vs. female subgroups.

A total of 13 studies (4, 5, 9, 10, 23, 24, 26–29, 31, 34, 39) reported the prevalence of CMD in post-PCI target vessels stratified by sex. Our meta-analysis showed that the pooled prevalence of CMD was 36.73% (95% CI: 28.17%–45.73%, *I*^2^ = 93.7%) in males and 46.22% (95% CI: 36.91%–55.66%, *I*^2^ = 91.1%) in females, with no statistically significant difference between subgroups (*P* = 0.14) (Figure 5).

**Figure 6:**
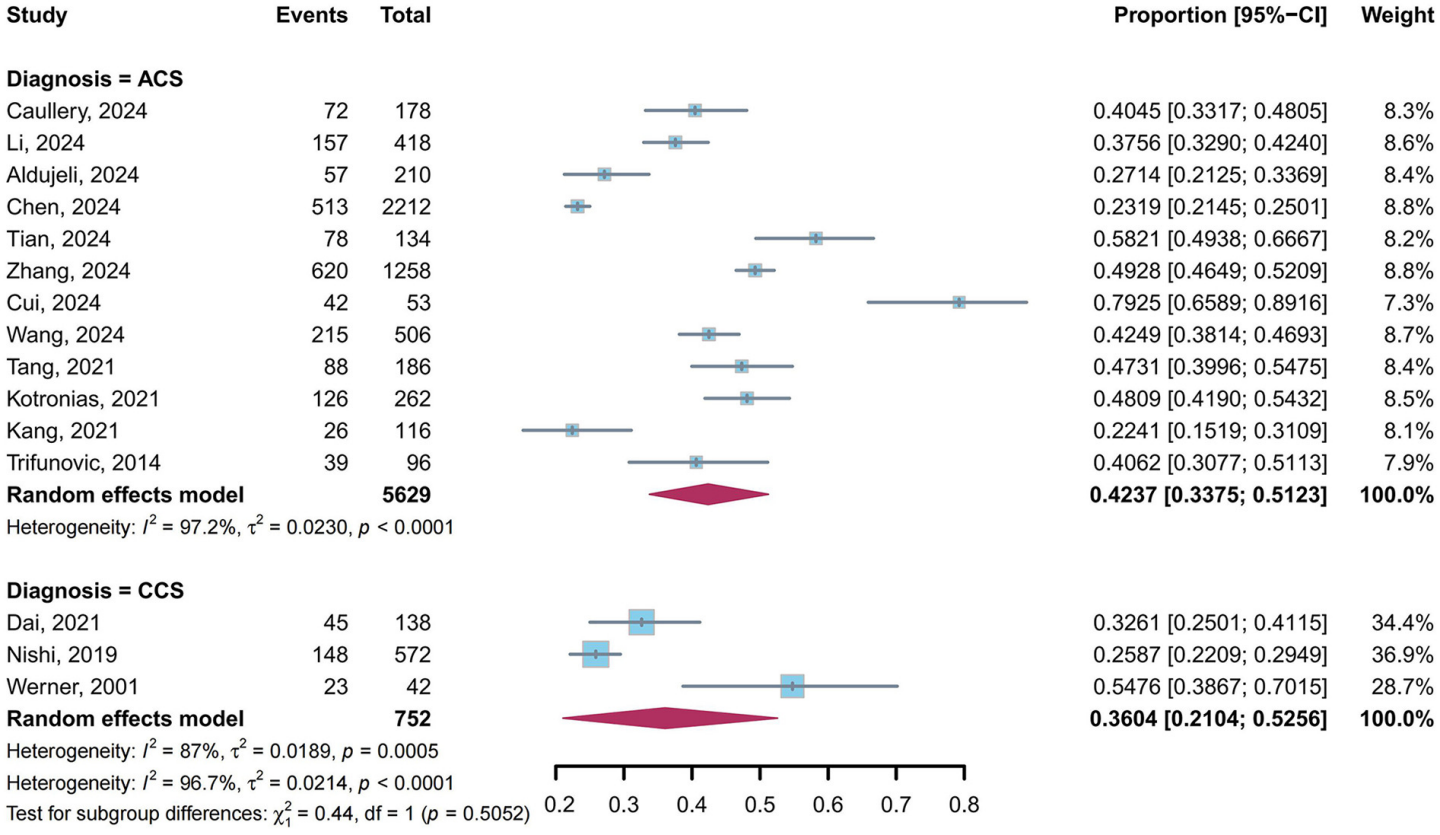
Prevalence of CMD in post-PCI target vessels: ACS vs. CCS subgroups.

A total of 15 studies (5, 9, 10, 23, 24, 26–29, 31, 32, 34–36, 39) reported the prevalence of CMD in post-PCI target vessels stratified by diagnosis: 12 studies (9, 23, 24, 26–29, 31, 34–36, 39) for ACS and 3 studies (5, 10, 32) for CCS. Our meta-analysis showed that the pooled prevalence of CMD in post-PCI target vessels was 42.37% (95% CI: 33.75%–51.23%, *I*^2^ = 97.2%) in ACS and 36.04% (95% CI: 21.04%–52.56%, *I*^2^ = 87%) in CCS, with no statistically significant difference between subgroups (*P* = 0.51) (Figure 6).

**Figure 7:**
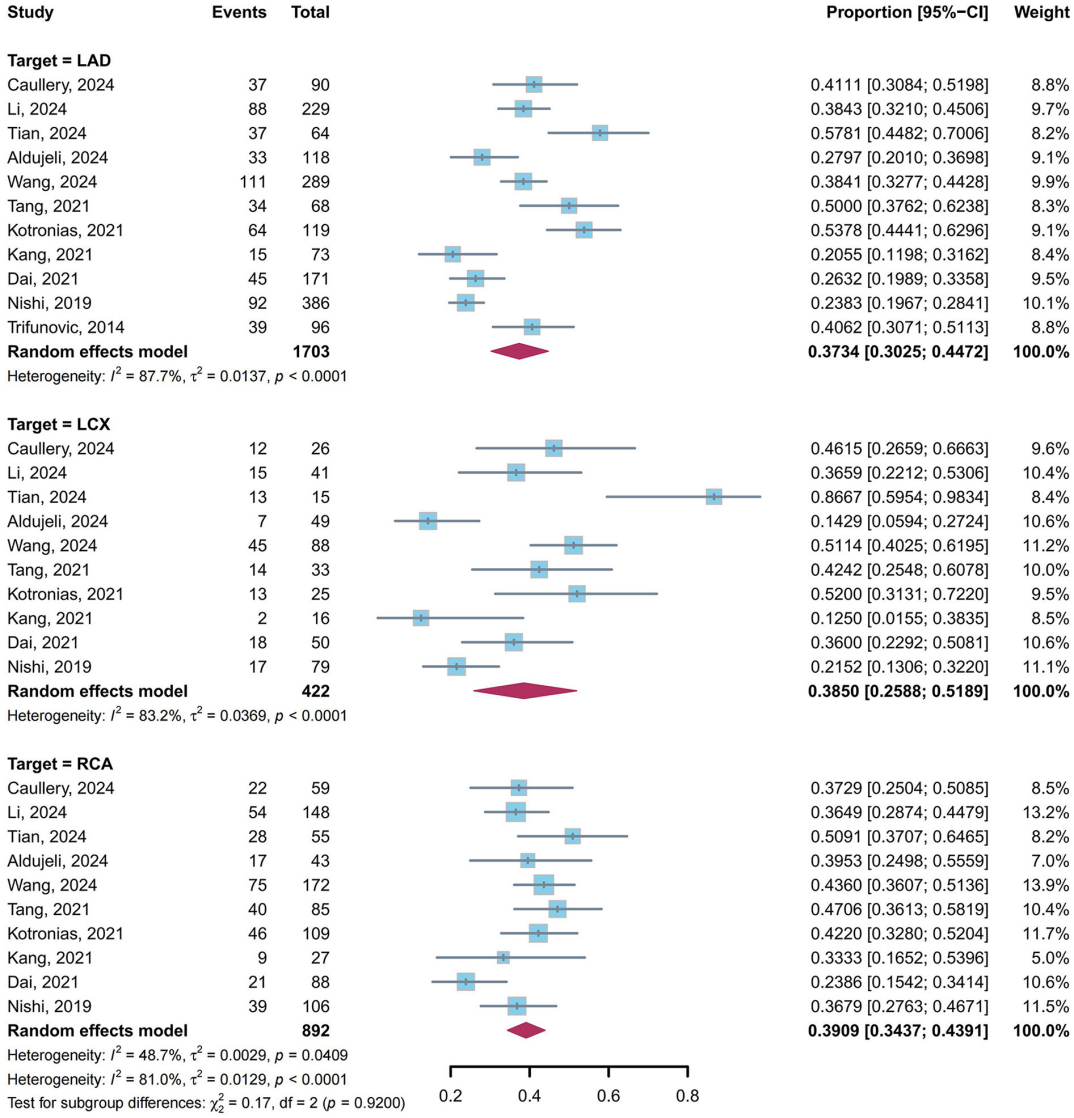
Prevalence of CMD in post-PCI target vessels: LAD vs. LCX vs. RCA subgroups.

A total of 11 studies (4, 5, 10, 23, 24, 26, 28, 29, 31, 34, 39) reported the prevalence of CMD in post-PCI target vessels stratified by target vessels: 11 studies (4, 5, 10, 23, 24, 26, 28, 29, 31, 34, 39) for LAD, 10 studies (4, 5, 10, 23, 24, 26, 28, 29, 31, 34) for LCX and 10 studies (4, 5, 10, 23, 24, 26, 28, 29, 31, 34) for RCA. Our meta-analysis showed that the pooled prevalence of CMD in post-PCI target vessels was 37.34% (95% CI: 30.25%–44.72%, *I*^2^ = 87.7%) in LAD, 38.50% (95% CI: 25.88%–51.89%, *I*^2^ = 83.2%) in LCX, and 39.09% (95% CI: 34.37%–43.91%, *I*^2^ = 48.7%) in RCA, with no statistically significant difference between subgroups (*P* = 0.92) (Figure 7).

**Figure 8:**
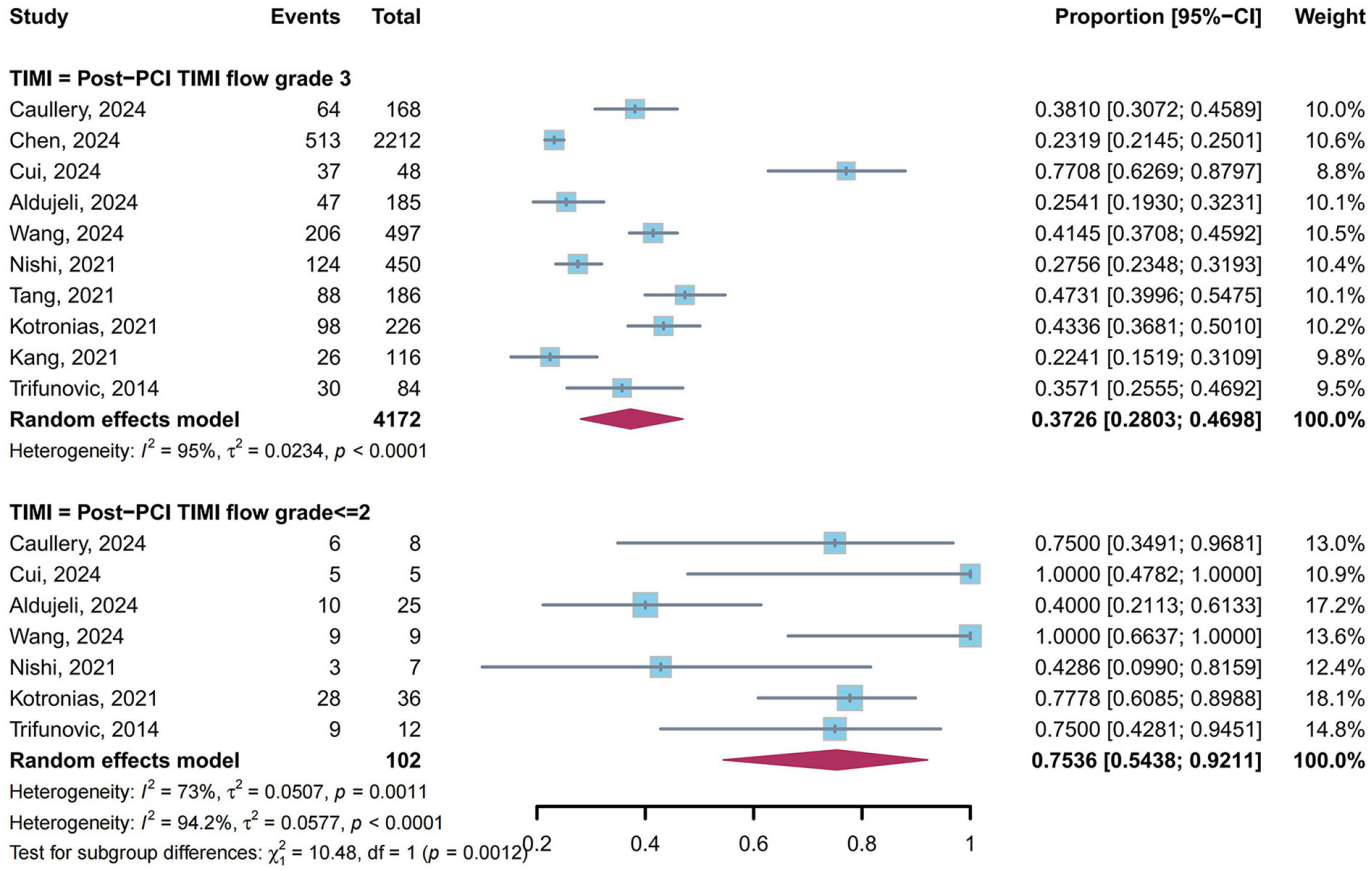
Prevalence of CMD in post-PCI target vessels: TIMI grade 3 vs. TIMI flow grade ≤2 subgroups.

A total of 10 studies (4, 9, 23–27, 31, 34, 39) reported the prevalence of CMD in post-PCI target vessels stratified by post-PCI TIMI flow grade: 10 studies (4, 9, 23–27, 31, 34, 39) for TIMI flow grade 3 and 7 studies (4, 9, 23, 25, 31, 34, 39) for TIMI flow grade ≤2. Our meta-analysis demonstrated that the pooled prevalence of CMD was 37.26% (95% CI: 28.03%–46.98%, *I*^2^ = 95%) in TIMI flow grade 3 and 75.36% (95% CI: 54.38%–92.11%, *I*^2^ = 73%) in TIMI flow grade ≤2, with a statistically significant difference between subgroups (*P* = 0.0012) (Figure 8).

#### 3.4.4 The clinical outcomes in patients with CMD in post-PCI target vessels

**Figure 9:**
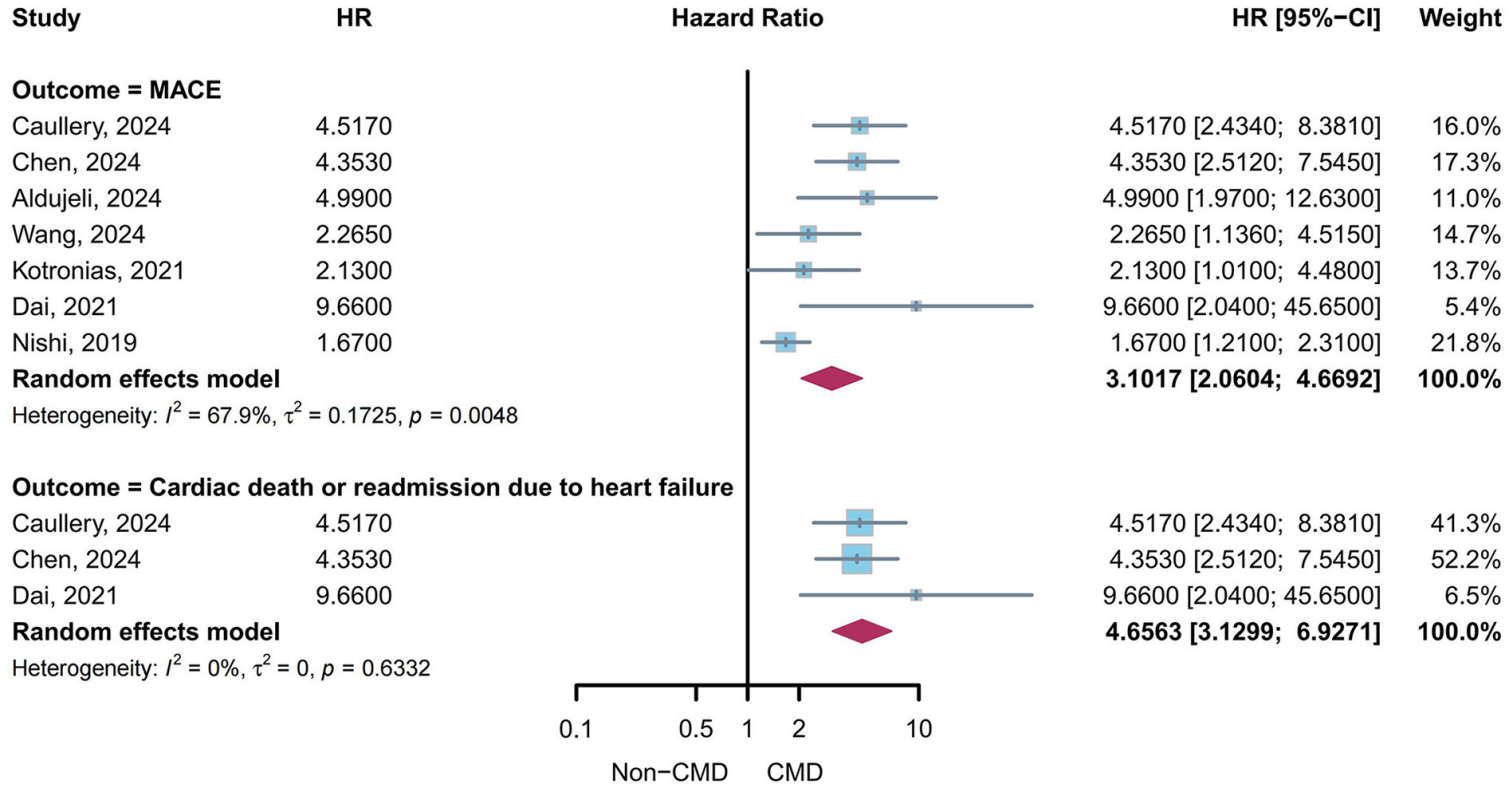
Pooled multivariable-adjusted HR for clinical outcomes in patients with CMD in post-PCI target vessels.

A meta-analysis of multivariable-adjusted HRs was performed to evaluate the association between CMD in post-PCI target vessels and clinical outcomes (Figure 9). The pooled HR for major adverse cardiovascular events (MACE) was 3.10 (95% CI: 2.06–4.67, *I*^2^ = 67.9%) based on data from 7 studies (4, 5, 10, 23, 27, 31, 34). For the composite outcome of cardiac death or heart failure readmission, the pooled HR was 4.66 (95% CI: 3.13–6.93, *I*^2^ = 0%) derived from 3 studies (5, 27, 34). Table 2 summarized the specific components of MACE in pooled study.

**Table 2:** The specific components of MACE in pooled studies.

Author	Region	Study design	MACE components	Follow-up
Caullery et al. (34)	France	Retrospective	cardiac death or readmission for heart failure	2.9 years (median)
Chen et al. (27)	China	Retrospective, multicenter	cardiac death or readmission for heart failure	2 years
Aldujeli et al. (4)	Lithuania	Prospective	cardiovascular death, nonfatal myocardial infarction, target vessel revascularization or stent thrombosis, recurrent hospitalization attributable to decompensated heart failure, and ischemic or hemorrhagic stroke	1 year
Wang et al. (23)	China	Retrospective	death from any cause, any myocardial infarction, readmission for heart failure, or any ischemia-driven revascularization	1 year
Kotronias et al. (31)	UK	Retrospective	all-cause mortality, resuscitated cardiac arrest and new heart failure diagnosis	4.2 years (median)
Dai et al. (5)	China	Retrospective, multicenter	cardiac death or readmission for heart failure	28 months (median)
Nishi et al. (10)	Australia, Belgium, Japan, USA	Retrospective, multicenter	all-cause mortality, any myocardial infarction, and target vessel revascularization	4 years (median)

MACE, major adverse cardiovascular events.

Outcomes unsuitable for meta-analysis due to insufficient data or methodological heterogeneity were described separately. Kang et al. (24) identified that high platelet-fibrin clot strength (P-FCS) (≥68 mm) significantly increased the risk of CMD [odds ratio (OR): 4.35, 95% CI: 1.74–10.89] post-PCI. Acute myocardial infarction (AMI) patients with both CMD and high P-FCS had a higher rate of MACE compared to non-CMD subjects with low P-FCS (OR: 5.58, 95% CI: 1.31–23.68). Chen et al. (27) demonstrated that the combination of diabetes mellitus (DM) and CMD in non-ST-elevation myocardial infarction (NSTEMI) patients was identified as the most powerful independent predictor of cardiac death or heart failure readmission at 2-year follow-up (adjusted HR: 7.894, 95% CI: 4.251–14.659, *P* < 0.001). Dai et al. (5) reported that in patients with CCS and CMD, the incidence of angina-related readmission was significantly higher during a median 28-month follow-up after the index procedure compared to CCS patients without CMD (46.7% vs. 19.4%, adjusted HR: 3.66, 95% CI: 1.68–7.97, *P* = 0.001). Aldujeli et al. (4) reported that in ST-elevation myocardial infarction (STEMI) patients with persistent CMD at 3 months post-PCI, those with CMD exhibited significantly poorer outcomes at 12-month follow-up compared to non-CMD counterparts. Specifically, CMD patients demonstrated markedly impaired recovery of left ventricular systolic function (−10.00% vs. +8.00%, *P* < 0.001), a higher prevalence of diastolic dysfunction (73.08% vs. 1.32%, *P* < 0.001), and elevated rates of adverse ventricular remodeling (11.32% vs. 7.28% and 22.64% vs. 1.99%, respectively, *P* < 0.001). Furthermore, IMR was independently associated with both left ventricular diastolic dysfunction and adverse remodeling (*P* < 0.001 for all associations).

## 4 Discussion

This systematic review and meta-analysis, encompassing 21 studies and 6,632 patients, consolidated the understanding that CMD in post-PCI target vessels is prevalent (41.66%, 95% CI: 34.18–49.34%) and independently associated with adverse clinical outcomes. Subgroup analyses revealed numerical variations in CMD prevalence across assessment methods, sex, clinical diagnoses, and target vessels, though intergroup differences did not reach statistical significance (all *P* > 0.05). Specifically, the pooled prevalence of CMD was numerically higher in females (46.22% vs. 36.73% in males), patients with ACS (42.37% vs. 36.04% in CCS), and those assessed via non-wire-based methods (44.72% vs. 35.65% in wire-based methods). CMD prevalence was comparable across target vessels (LAD: 37.34%, LCX: 38.50%, RCA: 39.09%), suggesting its nature irrespective of lesion location. Patients with suboptimal post-PCI TIMI flow (grade ≤2) exhibited a markedly higher prevalence of CMD (75.36%, 95% CI: 54.38%–92.11%) compared to those with TIMI grade 3 flow (37.26%, 95% CI: 28.03%–46.98%), with a statistically significant intergroup difference (*P* = 0.0012). Moreover, multivariable-adjusted meta-analysis demonstrated that CMD in post-PCI target vessels independently conferred a 3.10-fold increased risk of MACE (95% CI: 2.06–4.67) and a 4.66-fold risk of cardiac death or heart failure readmission (95% CI: 3.13–6.93), underscoring its prognostic significance.

Through technological advancements, CMD in target vessels after PCI has been substantiated as diagnosable via multiple coronary physiological assessments (15, 40). Among pressure wire-based methods, the index of microvascular resistance (IMR) and microvascular resistance reserve (MRR) specifically evaluate microvascular function independently of epicardial vessels. Conversely, assessments of CFR and coronary flow velocity reserve (CFVR) are confounded by epicardial blood flow and therefore must be conducted following PCI-mediated resolution of obstructive lesions in target vessels (41–43). Non-wire-based methods have garnered substantial attention owing to their procedural convenience and cost-effectiveness. Physiological coronary indices acquired through non-invasive techniques such as single photon emission computed tomography (SPECT) and transthoracic Doppler echocardiography (TDE) have demonstrated efficacy in delayed detection of post-PCI microvascular dysfunction in target vessels, yet remain inadequate for immediate intraprocedural identification of potential CMD (6). Importantly, despite potential influences from differing assumed boundary conditions (44), angiography-based microcirculation assessment techniques including angiography-derived microcirculatory resistance (AMR) have established diagnostic value for CMD. These techniques show strong correlation with IMR, the reference standard for specific coronary microvascular function evaluation (40), thereby enabling both immediate intraprocedural detection and subsequent management of CMD, along with epidemiological investigations leveraging historical coronary angiography data.

The marked heterogeneity in reported prevalence of post-PCI CMD (20%–80%) reflects not only differences in patient clinical characteristics but also methodological variability in diagnostic methods and study designs (6–10). Current coronary physiological assessments reveal that a substantial proportion of post-PCI CMD cases occur in vessels with angiographically normal flow, suggesting potential systematic underdiagnosis in studies relying on non-physiological assessments (13, 14). Furthermore, prior research often failed to distinguish between pre-existing non-target vessel CMD and post-PCI target vessel CMD, which likely conflates​distinct pathophysiological entities (16–18). To address these limitations, investigations were exclusively included in our study if they utilized quantitative coronary physiological assessments to evaluate CMD in post-PCI target vessels, thereby minimizing diagnostic variability and confounding from non-target vessel pathology. This rigorous approach, when combined with subgroup analyses, provides novel insights into the true burden and clinical implications of CMD in post-PCI target vessels.

Subgroup analyses provided nuanced insights into the distribution and mechanisms of CMD across different clinical contexts. In the assessment method subgroup, although no statistical difference was observed between non-wire-based and wire-based methods in CMD prevalence (44.72% vs. 35.65%, *P* = 0.30), the numerical disparity may reflect differences in technical applicability and sensitivity (45). Non-wire-based methods, especially those relying on angiographic image analysis, offer procedural simplicity and are suitable for emergency PCI but may be confounded by vasospasm or collateral circulation (23, 45). In contrast, wire-based methods directly measure coronary physiological parameters with higher precision but require invasive equipment and complex operations (46). It is worth emphasizing that most non-wire-based methods utilize angiography-derived microcirculatory assessment techniques, which have been validated to exhibit diagnostic accuracy comparable to the traditional wire-based gold-standard IMR measurements (40). These non-wire-based methods provide advantages in operational simplicity and enable dynamic assessment during PCI, potentially enhancing real-time detection of subtle microvascular dysfunction. Conversely, wire-based methods, while precise, are technically complex and costly, with practical limitations in routine clinical use, which may contribute to underdiagnosis of CMD in real-world settings (11).

In the sex subgroup analysis, the prevalence of CMD in post-PCI target vessels was numerically higher in females than in males (46.22% vs. 36.73%, *P* = 0.14). Although this difference did not reach statistical significance, the trend aligns with prior studies reporting sex-specific disparities in CMD (47, 48). The mechanisms underlying these differences remain incompletely understood, though evidence suggests that hormonal fluctuations during peri- and post-menopausal transitions may contribute to coronary endothelial dysfunction and abnormal vasomotor regulation (49, 50). Notably, prior studies demonstrated that myocardial ischemia in males predominantly originated from obstructive epicardial coronary disease, whereas in females it more frequently stemmed from non-obstructive coronary disease (51, 52). This resulted in female patients undergoing coronary angiography requiring PCI less frequently than their male counterparts. Consequently, pre-existing CMD in target vessels might be detected less often in females after PCI, potentially obscuring sex-based differences and contributing to underestimation of the overall prevalence.

The prevalence of CMD was numerically higher in ACS patients compared to CCS patients (42.37% vs. 36.04%, *P* = 0.51), consistent with studies linking ACS to microvascular dysfunction (11). This discrepancy may arise from ACS-specific mechanisms, including thrombotic lesion instability, ischemia-reperfusion injury, and lipid-rich plaque composition (11, 53). Intracoronary imaging studies have demonstrated that lipid-rich plaque morphology is independently associated with post-PCI microvascular dysfunction (11, 12). Mechanistically, lipid-core embolization during stent deployment may trigger distal microvascular obstruction and activate pro-inflammatory pathways, exacerbating endothelial injury and perpetuating microvascular dysfunction (6, 49). It should be noted that diagnostic criteria for CMD remain controversial across clinical subtypes of CAD. Based on the association with adverse long-term outcomes in prior studies, higher diagnostic thresholds than those used under general circumstances tend to be selected for immediate post-PCI diagnosis of STEMI-related CMD (54–56). This situation was prevalent in our included studies, potentially reducing the pooled prevalence in the ACS subgroup to some extent and indirectly leading to blurring of differences in post-PCI target-vessel CMD prevalence between ACS and CCS subgroups.

Notably, CMD prevalence exhibited consistency across target vessels (LAD: 37.34%, LCX: 38.50%, RCA: 39.09%, *P* = 0.92). Although the LAD supplies a larger myocardial territory and is associated with a well-developed coronary microcirculation (57, 58), the uniform distribution of CMD suggests that microvascular dysfunction post-PCI might occur independently of lesion location or vessel-specific anatomical characteristics.

Patients with TIMI flow grade ≤2 had a significantly higher CMD prevalence than those with grade 3 (75.36% vs. 37.26%, *P* = 0.0012), highlighting the clinical relevance of TIMI flow grade. This observation aligns with the pathophysiological mechanisms whereby suboptimal TIMI flow may reflect impaired myocardial perfusion secondary to microvascular obstruction or reperfusion injury (14, 59). Importantly, such perfusion deficits are strongly associated with adverse ventricular remodeling and progression to heart failure, thereby providing a mechanistic explanation for their established correlation with poor clinical outcomes (60).

Our meta-analysis suggested that CMD in post-PCI target vessels is an independent predictor of adverse clinical outcomes. The pooled multivariable-adjusted hazard ratios revealed a 3.10-fold increased risk of MACE and a 4.66-fold risk of the composite outcome of cardiac death or heart failure readmission in patients with CMD. These findings suggest that microvascular dysfunction post-PCI is a critical determinant of long-term prognosis. The heightened risk may stem from persistent myocardial ischemia, adverse ventricular remodeling, and endothelial dysfunction, which collectively exacerbate cardiovascular vulnerability even after successful revascularization of epicardial arteries. During the peri-PCI period, severe post-PCI CMD may cause significant impairment of microcirculatory perfusion in target vessels through mechanisms including mechanical obstruction induced by distal embolization of the epicardial coronary artery, external compression due to tissue edema, localized *in situ* thrombosis, vascular spasm, leukocyte stagnation, activation of inflammatory cascades, and reperfusion injury (61, 62). This may promote peri-procedural myocardial injury and even type 4a myocardial infarction, leading to further increases in cardiovascular adverse events (56, 63). Notably, studies such as Chen et al. (27) highlighted the synergistic effect of CMD and DM, amplifying the risk of cardiac death or heart failure readmission by nearly 8-fold. This underscores the importance of integrating CMD assessment into risk stratification paradigms.

Our study has several limitations. First, significant heterogeneity (*I*^2^ > 90%) existed across studies due to differences in diagnostic methods and study design, which may limit the generalizability of pooled estimates. Non-uniformity in detection details and timing was likely a primary source of heterogeneity, aligned with the inherent variability among current diagnostic modalities. Discrepancies in CMD diagnostic standards across clinical subtypes of CAD also contributed partially to heterogeneity, though this requires further research for clarification. Additionally, while the use of vasodilation induced by papaverine and continuous thermodilution might enhance the stability of microvascular measurements, the absence of these methods in the included studies could have increased heterogeneity (64–66). Second, the exclusive inclusion of observational studies utilizing quantitative coronary physiological assessments with prespecified diagnostic criteria, while enhancing diagnostic consistency, might have introduced selection bias by excluding populations where such evaluations were unavailable or clinically unfeasible. Third, despite comprehensive coverage of diagnostic subtypes of CAD, studies focusing on non-ST-elevation acute coronary syndrome (NSTE-ACS) remained underrepresented, potentially limiting insights into related subtypes. Fourth, all included studies explicitly reporting elective PCI were limited to patients with CCS, which may not fully reflect the broader clinical significance of elective PCI in other populations. Additionally, emergency PCI studies predominantly included cases of primary PCI for STEMI, which is not yet sufficient to further differentiate between primary PCI and rescue PCI post-thrombolysis. Consequently, our analysis did not provide subgroup results stratified by PCI type, which may limit the applicability of findings to specific clinical scenarios. Fifth, although we excluded CMD in non-target vessels, the data proved insufficient to distinguish between *de novo* post-PCI CMD and pre-existing CMD in target vessels because many studies lacked systematic pre-PCI CMD assessment. Sixth, the absence of standardized follow-up protocols and inconsistent reporting of MACE components complicated the interpretation of results. To scientifically and reliably assess the impact of post-PCI target vessel CMD on clinical outcomes while minimizing confounding effects from other factors, our study exclusively included and pooled HRs with 95% CIs that underwent multivariate adjustment. Given that most incorporated studies adjusted solely for MACE, this resulted in isolated outcomes that failed to meet the predefined pooling criteria of this study. Furthermore, even with multivariable adjustments, unmeasured confounders may persist, potentially influencing the observed associations between CMD and clinical outcomes. Finally, regarding outcomes, we did not analyze the impact of epicardial vascular functional parameters such as fractional flow reserve (FFR) on clinical outcomes. Given that our study primarily focused on the independent effect of post-PCI target vessel CMD on clinical outcomes, and considering that the direct association between epicardial vascular functional parameters and CMD remains unclear, we excluded this analysis from the present investigation.

## 5 Conclusion

This systematic review and meta-analysis demonstrated that CMD in post-PCI target vessels is prevalent (approximately 40%) and independently associated with a elevated risk of adverse cardiovascular outcomes, especially MACE and the composite outcome of cardiac death or heart failure readmission. Future efforts should focus on standardizing diagnostic criteria and developing tailored interventions to improve outcomes in this population.

## Data Availability

The raw data supporting the conclusions of this article will be made available by the authors, without undue reservation.
