# Supplementary material for: Coronary microvascular dysfunction in post-PCI target vessels: a systematic review and meta-analysis of prevalence and associated outcomes
Source: Front Cardiovasc Med. 2025 Aug 4;12:1620204. doi: 10.3389/fcvm.2025.1620204 (PMC12358399; doi:10.3389/fcvm.2025.1620204)
Supplement: Supplementary file 1 [file Table1.docx]

**Supplementary file**

**Content**

**Supplementary Table S1. Search strategy**

**Supplementary Table S2. Risk of bias assessments of the included studies**

**Supplementary figure 1. Funnel Plot of CMD in post-PCI target vessels prevalence.**

**1 Supplementary Figures and Tables**

**1.1 Supplementary Tables**

**Supplementary Table S1. Search strategy**

**PubMed**:

| (("Percutaneous Coronary Intervention"[Mesh]) OR (((((((((((((Coronary Intervention, Percutaneous[Title/Abstract]) OR (Coronary Interventions, Percutaneous[Title/Abstract])) OR (Intervention, Percutaneous Coronary[Title/Abstract])) OR (Interventions, Percutaneous Coronary[Title/Abstract])) OR (Percutaneous Coronary Interventions[Title/Abstract])) OR (Percutaneous Coronary Revascularization[Title/Abstract])) OR (Coronary Revascularization, Percutaneous[Title/Abstract])) OR (Coronary Revascularizations, Percutaneous[Title/Abstract])) OR (Percutaneous Coronary Revascularizations[Title/Abstract])) OR (Revascularization, Percutaneous Coronary[Title/Abstract])) OR (Revascularizations, Percutaneous Coronary[Title/Abstract])) OR (percutaneous coronary intervention[Title/Abstract])) OR (PCI[Title/Abstract]))) AND (((((((CMD[Title/Abstract]) OR (coronary microvascular dysfunction[Title/Abstract])) OR (coronary microvascular[Title/Abstract])) OR (coronary microcirculation[Title/Abstract])) OR (microvascular dysfunction[Title/Abstract])) OR (microcirculatory dysfunction[Title/Abstract])) OR (CMVD[Title/Abstract])) | 529 |
| --- | --- |

**Web of Science:**

| (((((((((((((TS=(percutaneous coronary intervention)) OR TS=(Coronary Intervention, Percutaneous)) OR TS=(Coronary Interventions, Percutaneous)) OR TS=(Intervention, Percutaneous Coronary)) OR TS=(Interventions, Percutaneous Coronary)) OR TS=(Percutaneous Coronary Interventions)) OR TS=(Percutaneous Coronary Revascularization)) OR TS=(Coronary Revascularization, Percutaneous)) OR TS=(Coronary Revascularizations, Percutaneous)) OR TS=(Percutaneous Coronary Revascularizations)) OR TS=(Revascularization, Percutaneous Coronary)) OR TS=(Revascularizations, Percutaneous Coronary)) OR TS=(PCI)) AND (((((((TS=(coronary microvascular dysfunction)) OR TS=(coronary microvascular)) OR TS=(coronary microcirculation)) OR TS=(microvascular dysfunction)) OR TS=(microcirculatory dysfunction)) OR TS=(CMD)) OR TS=(CMVD)) | 1935 |
| --- | --- |

**Embase:**

| ('percutaneous coronary intervention'/exp OR 'percutaneous coronary intervention':ti,ab,kw OR 'coronary intervention, percutaneous':ti,ab,kw OR 'coronary interventions, percutaneous':ti,ab,kw OR 'intervention, percutaneous coronary':ti,ab,kw OR 'interventions, percutaneous coronary':ti,ab,kw OR 'percutaneous coronary interventions':ti,ab,kw OR 'percutaneous coronary revascularization':ti,ab,kw OR 'coronary revascularization, percutaneous':ti,ab,kw OR 'coronary revascularizations, percutaneous':ti,ab,kw OR 'percutaneous coronary revascularizations':ti,ab,kw OR 'revascularization, percutaneous coronary':ti,ab,kw OR 'revascularizations, percutaneous coronary':ti,ab,kw OR pci:ti,ab,kw) AND ('coronary microvascular dysfunction'/exp OR cmd:ti,ab,kw OR 'coronary microvascular dysfunction':ti,ab,kw OR 'coronary microvascular':ti,ab,kw OR 'coronary microcirculation':ti,ab,kw OR 'microvascular dysfunction':ti,ab,kw OR 'microcirculatory dysfunction':ti,ab,kw OR cmvd:ti,ab,kw) | 1188 |
| --- | --- |

**Supplementary Table S2. Risk of bias assessments of the included studies**

| **Study ID** | **Was the sample frame appropriate to address the target population?** | **Were study participants recruited in an appropriate way?** | **Was the sample size adequate?** | **Were the study subjects and setting described in detail?** | **Was data analysis conducted with sufficient coverage of the identified sample?** | **Were valid methods used for the identification of the condition?** | **Was the condition measured in a standard, reliable way for all participants?** | **Was there appropriate statistical analysis?** | **Was the response rate adequate, and if not, was the low response rate managed**  **appropriately?** | **Overall score** |
| --- | --- | --- | --- | --- | --- | --- | --- | --- | --- | --- |
| Caullery, 2024 | Y | Y | Y | Y | Y | Y | Y | Y | U | 8 |
| Li, 2024 | Y | Y | Y | Y | Y | Y | Y | Y | NA | 8 |
| Aldujeli, 2024 | Y | Y | Y | N | Y | Y | Y | Y | U | 7 |
| Chen, 2024 | Y | Y | Y | Y | Y | Y | Y | Y | Y | 9 |
| Tian, 2024 | Y | Y | Y | Y | Y | Y | Y | Y | Y | 9 |
| Zhang, 2024 | Y | Y | Y | N | Y | Y | Y | Y | NA | 7 |
| Cui, 2024 | Y | Y | N | Y | Y | Y | Y | Y | NA | 7 |
| Tsai, 2024 | Y | Y | Y | Y | Y | Y | Y | Y | U | 8 |
| Aldujeli, 2024 | Y | Y | Y | Y | Y | Y | Y | Y | U | 8 |
| Wang, 2024 | Y | Y | Y | Y | Y | Y | Y | Y | Y | 9 |
| Aldujeli, 2023 | Y | Y | Y | N | Y | Y | Y | Y | U | 7 |
| Nishi, 2021 | Y | Y | Y | Y | Y | Y | Y | Y | U | 8 |
| Tang, 2021 | Y | Y | Y | Y | Y | Y | Y | Y | Y | 9 |
| Kotronias, 2021 | Y | Y | Y | Y | Y | Y | Y | Y | Y | 9 |
| Kang, 2021 | Y | Y | Y | N | Y | Y | Y | Y | U | 7 |
| Dai, 2021 | Y | Y | Y | Y | Y | Y | Y | Y | U | 8 |
| Yang, 2019 | Y | Y | N | N | Y | Y | Y | Y | NA | 6 |
| Nishi, 2019 | Y | Y | Y | Y | Y | Y | Y | Y | U | 8 |
| Trifunovic, 2014 | Y | Y | N | Y | Y | Y | Y | Y | NA | 7 |
| Werner, 2001 | Y | Y | N | N | Y | Y | Y | Y | NA | 6 |
| Qian, 1999 | Y | Y | Y | N | Y | Y | Y | Y | NA | 7 |

Y: yes; N: no; U: unclear; NA: not applicable

**1.2 Supplementary Figures**

**
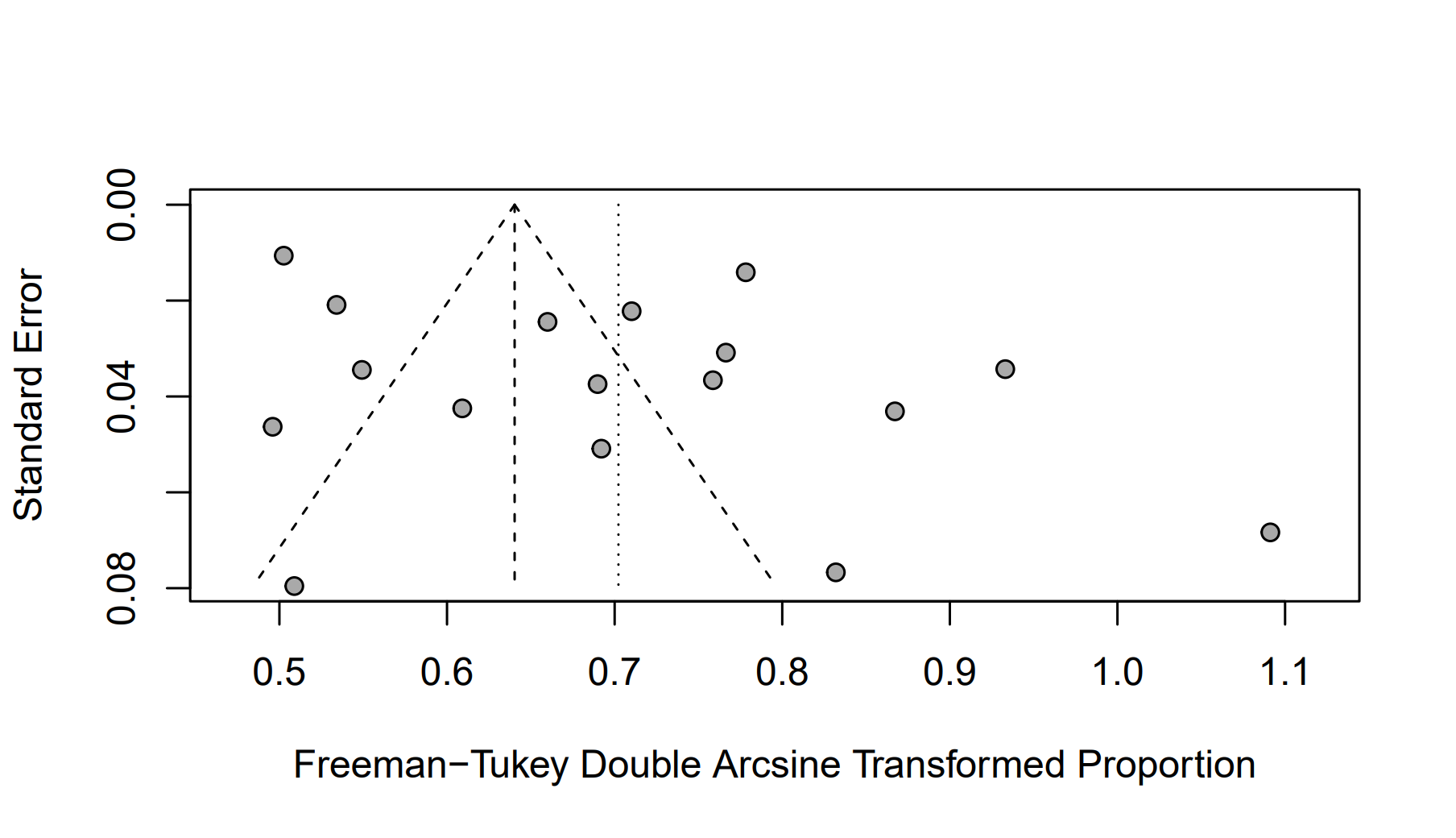
**

**Supplementary figure 1. Funnel Plot of CMD in post-PCI target vessels prevalence.**
